# Response to Anti-PD-1 Therapy in Metastatic Merkel Cell Carcinoma Metastatic to the Heart and Pancreas

**DOI:** 10.7759/cureus.403

**Published:** 2015-12-13

**Authors:** Kalyan Mantripragada, Ariel Birnbaum

**Affiliations:** 1 Rhode Island Hospital, The Warren Alpert Medical School of Brown University

**Keywords:** immune, merkel cell carcinoma, polyoma, pd-1, pd-l1, nivolumab, t cells, cd8, til, checkpoint

## Abstract

Metastatic Merkel cell carcinoma (MCC) is a lethal, Merkel cell polyomavirus (MCPyV) cancer with no currently available effective therapy. Harnessing the immune system through an immune checkpoint blockade is an attractive option because the immune system appears to be dysfunctional in the Merkel cell tumor microenvironment. Although MCPyV is expressed in 80% of MCCs and serves as a powerful antigen for stimulating host immune response, intratumoral CD8+ T-cell infiltration is seen only in 18% of MCCs. In contrast, about 50% of MCPyV-positive MCCs express the programmed death-ligand 1 (PD-L1) on multiple cell types in the tumor microenvironment. We present a case of a young patient with MCC involving the heart and pancreas that showed an impressive response after treatment with four cycles of the anti-PD-1 monoclonal antibody, nivolumab.

## Introduction

Merkel cell carcinoma (MCC) is an aggressive, Merkel cell polyomavirus (MCPyV)-driven, cutaneous neuroendocrine cancer with increasing incidence. The metastatic disease develops in a significant number of patients and confers a three-year survival rate of 20% [1]. Available chemotherapeutic agents do not prolong survival. Therefore, newer therapeutic options are urgently needed.

The immune system is dysfunctional in the MCC tumor microenvironment. MCC is often seen in elderly and immunosuppressed patients. Although MCPyV oncoproteins are capable of inducing anti-tumor immunity, such responses are confined to a minor percentage of tumors [2]. In addition, about half of the MCPyV-positive MCCs express the immunosuppressive programmed death ligand-1 (PD-L1) on tumor cells and cells in the tumor microenvironment [3]. PD-L1 is a ligand for a programmed death-1 (PD-1) surface receptor, which is expressed by activated T lymphocytes. Ligation of PD-1 with PD-L1 inhibits the anti-tumor immune response. We report a case of a patient with MCC metastases to the heart and pancreas, who obtained an impressive response to the anti-PD-1 monoclonal antibody, nivolumab (BMS-936558; Bristol-Myers Squibb, NY, USA).

## Case presentation

The patient is a 42-year-old Hispanic man with a history of testicular cancer for which he underwent orchiectomy, adjuvant radiation therapy, and chemotherapy while in his twenties. Details of this treatment could not be obtained. He has no other medical history and no family history of cancer. He initially presented at the age of 40 with a palpable mass in the right submandibular area. Computed tomography (CT) showed a 2.5 x 1.4 cm submandibular lymph node and a needle biopsy revealed Merkel cell carcinoma (MCC). There were no other suspicious lesions on examination of his skin, laryngoscopy, and whole body positron emission tomography (PET). The patient underwent a selective neck dissection with metastases in three of the five lymph nodes examined and no extranodal spread (TX N1 M0, Stage IIIB, according to America Joint Commission on Cancer Staging Manual, seventh edition, 2010). He then received adjuvant intensity-modulated radiation therapy (IMRT) to a total dose of 59.4 Gy (daily dose of 180 cGy per fraction; 33 fractions) followed by four cycles of adjuvant chemotherapy with cisplatin at 80 mg/m^2^ on day 1 and etoposide, 100 mg/m^2^, on days 1, 2, and 3 of a 21-day cycle. Treatment was complicated by a Grade II peripheral neuropathy as per Common Terminology Criteria for Adverse Events, version 4.0.

**Figure 1:**
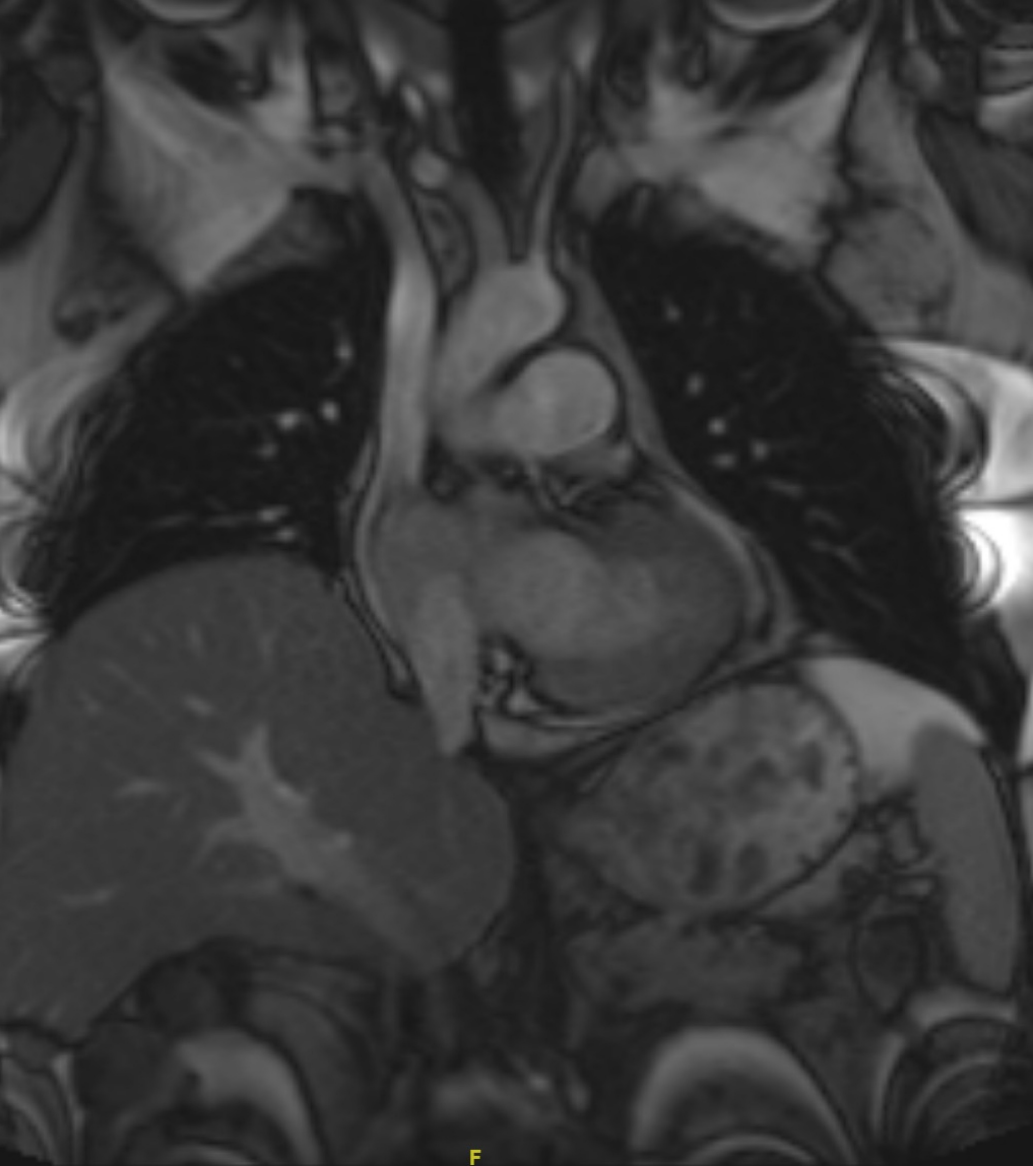
Cardiac Involvement with Merkel Cell Carcinoma Cardiac MRI showing involvement of the myocardium with Merkel cell carcinoma.

One year after his diagnosis, the patient presented with abdominal pain. Laboratory tests showed a lipase of 767 IU/L, aspartate aminotransferase of 281 IU/L, alanine aminotransferase of 372, and alkaline phosphatase of 314 IU/L. Magnetic resonance imaging (MRI) of the abdomen revealed intrahepatic and extrahepatic biliary dilatation, a soft tissue mass within the right cardiac ventricle, and a prominent pericardial lymph node. Further evaluation with a cardiac MRI (Figure 1) revealed two discrete enhancing masses within the heart—one measuring 3.5 x 3.0 cm along the inferior right ventricular apical free wall with intra- and extracardiac extension, and the second measuring 2.4 x 2.7 cm within the posterior intra-atrial septum. In addition, there was an 11 mm pericardial lymph node adjacent to the left ventricle. The patient’s lipase and liver function tests normalized with conservative management of pancreatitis. A whole body PET scan revealed intense 18-flurodeoxyglucose (FDG) activity corresponding to the intracardiac masses and the pericardial lymph node. In addition, two adjacent foci of intense FDG activity within the pancreatic head (2.0 x 2.0 cm and 2.7 x 2.0 cm) were seen, consistent with metastatic disease. Ultrasound-guided biopsy of the pancreatic lesion proved metastatic MCC. Cardiac function was normal.

**Figure 2:**
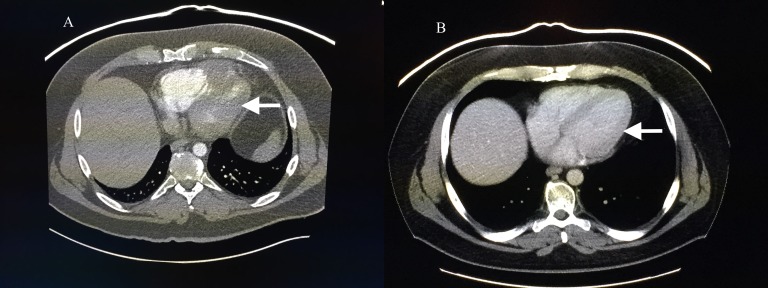
Response to Nivolumab CT scan of the chest showing extent of cardiac involvement (A) before and (B) after nivolumab therapy.

After discussing available options and obtaining informed patient consent, the patient was started on off-label therapy with nivolumab, 3 mg/kg intravenously every two weeks, which was made available through a patient assistance program. Two weeks after completion of the fourth cycle of nivolumab, CT showed a marked reduction in tumor burden. The right ventricular apical mass measured 1.5 x 0.9 cm and the mass in the intra-atrial septum was no longer visualized (Figure 2). One of the two pancreatic head masses disappeared and the other was ill-defined and measured 0.9 cm. The patient’s pain resolved and he continues on nivolumab without any significant adverse events. 

## Discussion

There are several interesting findings in this case. MCC predominantly affects elderly, fair-skinned individuals, and from that point of view, our patient was unusual. The risk of MCC is increased in patients with a history of other malignancies, although it typically appears within one year of the prior tumor. This patient presented initially with a head and neck MCC of occult primary, and in these cases, the primary tumor is believed to have undergone an immune-mediated spontaneous regression. Finally, cardiac metastases from MCC are rarely reported but can be seen in 12% of patients on autopsy [4].

The young age of the patient, unclear survival benefit with palliative chemotherapy in metastatic MCC, history of prior chemotherapy and RT, and the presence of peripheral neuropathy served as a rationale behind exploring alternative treatment options when the patient developed metastatic disease. Because MCC may be driven by an oncogenic virus, immunotherapy with PD-1 antibody was thus chosen as an alternative. MCPyV is associated with > 80% of MCC cases and aids in malignant transformation by clonal integration into the tumor cells. The dynamic interplay between MCPyV and the host immune system seems to control the natural history of MCC. Patients with high serum antibody titers against MCPyV oncoproteins and whose MCC harbors CD3+ and CD8+ tumor-infiltrating lymphocytes (TILs) demonstrate improved survival [2]. However, a significant immune dysfunction is present in a majority of MCCs because such TILs are limited to 18% of the tumors. In addition, about half of MCPyV-positive MCCs express PD-L1 on tumor cells, lymphocytes, and histiocytes present in a tumor microenvironment [3]. PD-L1 expression and TILs geographically co-localize, suggesting an immune evasion system that can be therapeutically exploited using the PD-1/PD-L1-targeting immune checkpoint inhibitors. Our case demonstrates the successful use of nivolumab to treat metastatic MCC in a patient unselected for PD-L1 expression or MCPyV positivity. Recently, Nghiem, et al. reported interim results of a Phase II trial studying another PD-1-directed antibody, pembrolizumab, as the first systemic therapy in patients with cutaneous MCC [Meeting abstract: LBA22, 2015 European Cancer Congress, September 25-29, Vienna, Austria]. Among 18 patients who received at least one dose of pembrolizumab, 10 patients had radiographically evaluable disease. Of these 10 patients, eight patients had shown evidence (five confirmed and three unconfirmed) of response to the PD-1 pathway blockade. Three of three additional patients with clinically evaluable metastases had clinical regression of tumors prior to their first scans. One patient had a Grade 4 myocarditis after one dose, and one had a Grade 4 transaminase elevation after two doses, with improvement in both patients after discontinuation of study drug and steroid administration. An ongoing open-label, Phase 1/2 study is assessing the role of nivolumab in subjects with virus-associated tumors (clinicaltrials.gov identifier NCT02488759). The differential benefit of immune checkpoint blockade according to MCPyV status in MCC is currently unknown.

## Conclusions

Metastatic MCC is a lethal cancer with no standard effective treatment. Harnessing the immune system by an immune checkpoint blockade is an attractive option, given the marked immune dysfunction seen in the tumor microenvironment of MCC. Our case stands as an example of exceptional responses seen with anti-PD-1 therapy in this disease. Future strategies include identification of patient and tumor characteristics that predict response to an immune checkpoint blockade. 
